# How Resilience Promotes Mental Health of Patients With DSM-5 Substance Use Disorder? The Mediation Roles of Positive Affect, Self-Esteem, and Perceived Social Support

**DOI:** 10.3389/fpsyt.2020.588968

**Published:** 2020-12-03

**Authors:** Chunyu Yang, You Zhou, Mengfan Xia

**Affiliations:** ^1^College of Law and Political Science, Nanjing University of Information Science and Technology, Nanjing, China; ^2^School of Social and Behavioral Sciences, Nanjing University, Nanjing, China; ^3^The Graduate School of Humanities and Social Science, University of Melbourne, Melbourne, VIC, Australia

**Keywords:** substance use disorders, resilience, mental health, positive affect, self-esteem, perceived social support

## Abstract

**Objectives:** The existing studies found that resilience is a salient trait that can significantly affect people's psychological well-being with substance use disorders (SUDs). However, few studies examined how the mechanisms are connected between resilience and mental health among patients with Diagnostic and Statistical Manual of Mental Disorders—fifth edition SUD. This study investigated the mediation effects of positive affect, perceived social support, and self-esteem on the effect of resilience on perceived stress and life satisfaction in SUD patients.

**Design:** A total of 415 patients diagnosed with Diagnostic and Statistical Manual of Mental Disorders—fifth edition SUD from the south of China joined the research.

**Outcome Measures:** The study applied Connor–Davidson Resilience Scale, Positive and Negative Affect Scale, Multidimensional Scale of Perceived Social Support, Rosenberg Self-Esteem Scale, and Satisfaction with Life Scale to measure patients' resilience, positive affect, self-esteem, perceived social support, perceived stress, and life satisfaction.

**Results:** Structural equation model analysis revealed that positive affect and self-esteem partially mediate the relationship between resilience and perceived stress. In contrast, positive affect and perceived social support partially mediate the relationship between resilience and life satisfaction.

**Conclusion:** The findings provide insights for evidence-based substance abuse intervention that positive affect, self-esteem, and perceived social support can conditional the effects of resilience on promoting the mental health of SUD patients.

## Background

Substance use disorders (SUDs) have been widely considered a global threat, posing enormous risks to individual well-being and cohesion of societies (1). As one of the overarching social problems, numerous studies have examined the correlations between SUD and psychological processes. Many studies show that perceived stress and life satisfaction are two prominent factors that play significant roles in influencing addiction severity and integral well-being (2).

Perceived stress is defined as individuals' cognitive appraisals over their stress level (3). Numerous addiction theories have depicted the theoretical linkage between perceived stress and substance abuse (4–6). For example, *tension reduction theory* (6) and self-medication hypothesis (5) postulate that chronic perceived stress is one of the primary motivations of taking illicit substances, which may temporarily alleviate psychological distress. Empirical findings also reveal that exposure to stress and post-traumatic stress disorder can increase illicit drug consumption level, frequency, and severity (7–11). Further, clinical observations suggest that substance users with higher perceived stress tend to report higher relapse rates (12, 13). Therefore, exploring the strategies to reduce perceived stress among people with SUD is of great importance for minimizing SUD's detrimental impacts and enlarging both the physical and mental well-being of people with SUD.

Life satisfaction is conceptualized as an individual's cognitive appraisal regarding their life's overall satisfaction (14). Life satisfaction has been used as a complementary method for assessing psychological well-being (15). Wide ranges of studies suggest that low life satisfaction is one of the salient predictors of some psychological disorders, including SUDs (2), anxiety (16), depression (17), and internet addiction disorders (18). Individuals with high life satisfaction report a lower level of substance abuse and mental health disorders (16). Life satisfaction has also been implemented as a diagnostic tool for SUD rehabilitation (19). Thus, exploring the potential mechanisms that boost life satisfaction among patients with SUD is crucial for combating the physical and psychological distress, promoting SUD rehabilitation processes.

## Resilience

Resilience has been regarded as one of the most critical determinants closely correlated to perceived stress and life satisfaction (20, 21). The definitions of resilience are generally based around two concepts, adversities and positive adaptions, conceptualizing individuals' capacity to bounce back when exposed to ranges of misfortunes (22, 23). The recent studies tend to define resilience, beyond the scope of a trait, as a dynamic psychological process that is susceptible to demographic factors [e.g., population, time, and place; (24)] and tends to promote other psychological traits [e.g., affect balance, self-esteem, and perceived social support; (23, 25)].

The relationships among resilience, perceived stress, and life satisfaction have been well-documented. A substantial body of studies conducted in the populations of non-users have shown that individuals with a higher level of resilience are reported to have lesser perceived stress in life events (20, 26, 27). Studies also suggest that people with higher resilience tend to experience a higher level of life satisfaction (28). However, although theoretical and empirical studies have suggested the associations among resilience, perceived stress, and life satisfaction, few focused on exploring the underlying mechanisms among them, especially among patients with SUD.

## Positive Affect, Self-Esteem, and Perceived Social Support as Mediators

Based on the existing theoretical and empirical studies, three items were the potential mediators in the impacts of resilience on perceived stress and life satisfaction. The first potential mediator is positive affect, whose definition was distinguished from positive emotion in history, but both have been used interchangeably nowadays (29, 30). Positive affect is conceptualized as the “pleasant ends” that can produce adaptive outcomes for flourishing individuals' mental and physical health (29). Fredrickson (31) suggests that positive affect is encompassed by the *broaden-and-build theory* (31–33), which denotes that positive affect can broaden mindsets by building enduring bio-psycho-social resources (e.g., social connections and coping methods), then achieving long-term adaptive outcomes such as happiness (34), psychological growth (35), creativity (36), immune function (37), reduction of an inflammatory response (38), and physical pain release (39). Studies have demonstrated that positive affect has an interactive relationship with resilience (40, 41) and life satisfaction (42, 43). Meanwhile, studies also identify that positive affect can significantly and effectively buffer the adverse impacts of perceived stress by widening thought–action repertoires, which facilitate generativity and behavioral flexibility (44). Based on theoretical and empirical studies, we hypothesized that positive affect would be the first mediator in the present study.

Extant studies indicate that self-esteem may be the second mediator. The widely accepted definition of self-esteem refers to the individual's general evaluation toward themselves (45, 46). However, there is a dispute regarding whether self-esteem is a component of resilience, the present study aligned with the mainstream perspectives which treat self-esteem as a separate concept (47–50). People with high self-esteem are motivated to maintain positive evaluations of themselves, further denoted by *terror management theory* (51). The theory depicts that self-esteem works as a buffer for anxiety-related events and various threats, promoting and maintaining a positive self-evaluation [e.g., (45, 51)].

Numerous empirical studies have shown that resilience can significantly facilitate self-esteem (25, 48, 52). Meanwhile, self-esteem is reported as a strong predictor of life satisfaction (45, 53, 54). Although rare attention has been paid to the association between self-esteem and perceived stress (55, 56), the terror management theory (51) posits that self-esteem is beneficial for preventing individuals from the impacts of the anxiety-related event, a salient predictor of perceived stress (57). Thus, the present study hypothesized that self-esteem is the second mediator in the impacts of resilience on perceived stress and life satisfaction in SUD patients.

Perceived social support is identified as the third mediator of the links. Although there is a statement that a supportive relationship is a key refinement of resilience, Pangello et al. (58) suggest that further research with regard to the overlaps between resilience and other concepts is needed, as the definitions and operationalizations of resilience are inconsistent. Therefore, the current research regarded perceived social support as an independent concept. Perceived social support reflects the individual's judgment over the general availability of support from relational and social boundaries (59).

The relationship between resilience and perceived social support has been documented by a wide range of studies (60–62). A majority of research focuses on examining the effects of perceived social support on resilience, suggesting that individuals with high perceived social support are reported with a higher level of resilience (60, 61, 63). A small group of studies explored how resilient people broaden their social networks and acquire supports from the established network. For example, Sexton et al. (64) suggested that resilient people are more likely to express their thoughts and find sympathetic friends, which are the salient factors for reducing psychological burdens (62). Furthermore, perceived social support has been identified with the roles of maintaining physical and mental well-being (65–69). Notably, groups of studies have found that people with high perceived social support are reported with a higher level of life satisfaction (70, 71), whereas some studies suggest that perceived social support has a negative association with perceived stress (72, 73). Based on the present observations, we hypothesized that perceived social support is the third mediator of the study.

## Context of Substance Use Disorders

Many social studies emphasize that psychological processes are susceptible to contextual and situational factors (74, 75). SUD is one of the significant contextual factors that can contribute to a wide range of variances in an individual's biological homeostasis (76, 77), psychological states (78, 79), relational and social boundaries (80), occupational performance (81), and cultural beliefs (82).

The present study focused on examining how underlying associations are shaped between psychological traits and mental health within the context of SUD. People with SUD may suffer more physical, psychological, relational, and social difficulties and challenges than non-users. For example, due to discrimination and social exclusion, studies showed that people with SUD report a lower level of perceived social support than non-users (83). Also, the relationship between support-giver and patients has been altered by bio-power, formed through designated interventions (59). Therefore, how the psychological traits are associated with mental health within the context of SUD is uncertain.

## Present Study

**Figure 1:**
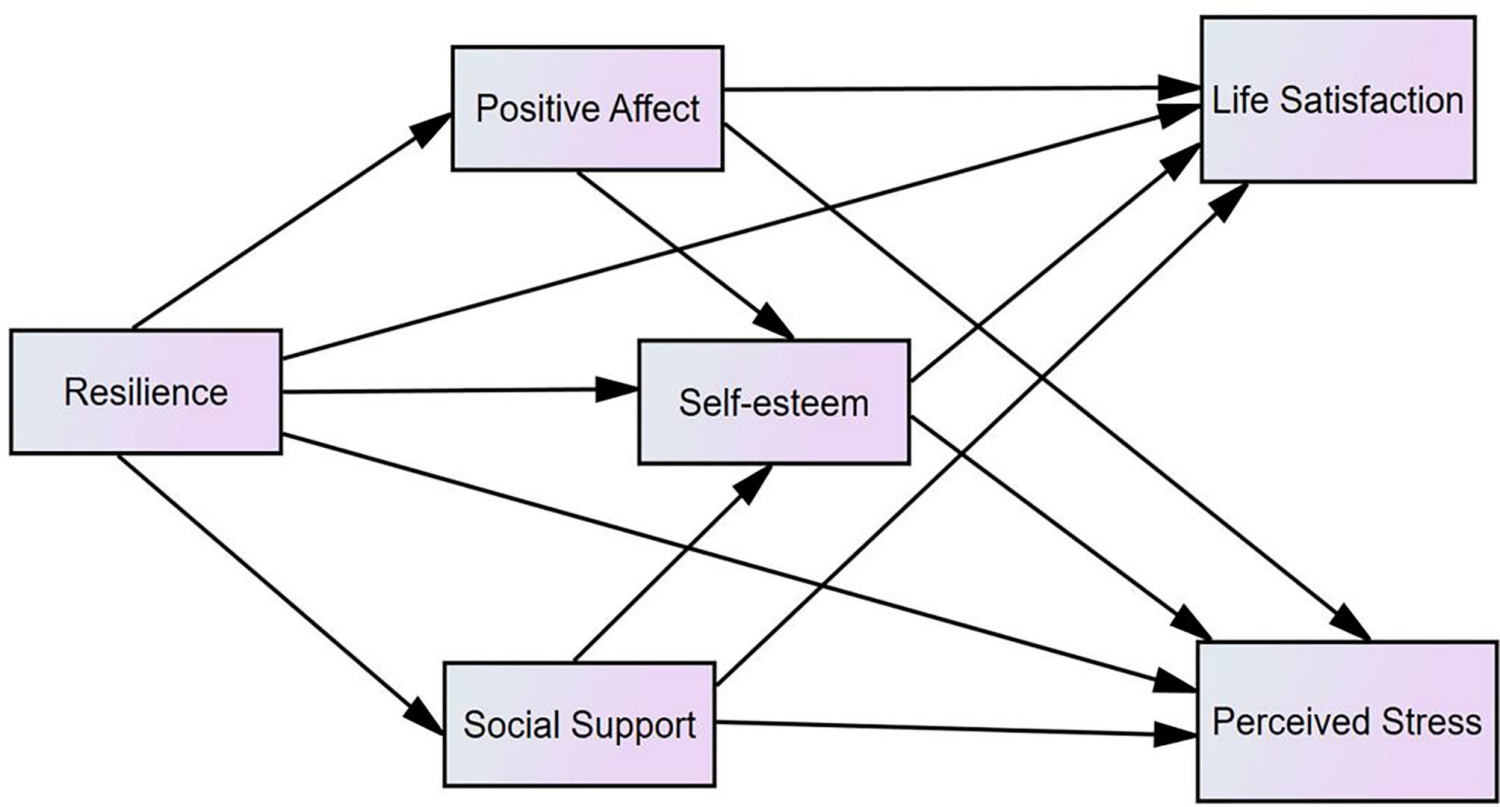
The hypothesized model (*N* = 415).

To reveal the uncertainties, the present study dedicated to examining whether positive affect, self-esteem, and perceived social support mediate the effects of resilience on perceived stress and life satisfaction, respectively. Based on the previous studies, we hypothesized that resilience exerts effects on perceived stress and life satisfaction via positive affect, self-esteem, and social support among people with SUD (Figure 1).

## Methods

### Participants and Design

The participants were comprised of 415 (322 males and 93 females, excluding one missing data) patients with SUD. All participants were recruited from two rehabilitation centers in the south of China. Nine demographic characteristics were measured in the study: age, sex, education, marital status, annual income, employment, months of detoxification, and substance types. The inclusion criteria of the study were as follows: age of 18 years or more, right-handed, normal color perception, regular and stable cognition, diagnosed with SUD within the last 12 months, and the voluntary willingness to participate. The exclusion criteria included: cognitive disabilities, psychiatric impairments, and a history of acute heart, kidney, and liver diseases, and the unwillingness to participate. As there were few missing values, we adopted listwise deletion for the cases with more than two missing values and mean imputation for the cases that had one missing value. The details of the demographic characteristics of 415 participants are shown in Table 1.

**Table 1:** Sample characteristics.

**Sample characteristics**		**Total (*****N*** **= 415)**		**Male**		**Female**	
		**M**	**SD**	***n***	**%**	***n***	**%**
Age (20–61 years)	Male	39.17	9.19	–	–	–	–
	Female	36.18	8.94	–	–	–	–
		***n***	**%**				
Gender	(1) Male	322	77.6	–	–	–	–
	(2) Female	93	22.4	–	–	–	–
Education: (*n* = 404)	(1) Elementary school and below	76	18.3	64	19.9	12	12.9
	(2) Middle school	222	53.5	166	51.6	56	60.2
	(3) High school	78	18.8	63	19.6	15	16.1
	(4) College and above	28	6.7	18	5.6	10	10.8
Marital status: (*n* = 410)	(1) Single	128	30.8	98	30.4	30	32.3
	(2) Married	140	33.7	106	32.9	34	36.6
	(3) Divorced	132	31.8	105	32.6	27	29.0
	(4) Widowed	10	2.4	8	2.5	2	2.2
Annually income(yuan/year): (*n* = 402)	<10,000	106	25.5	67	20.8	39	41.9
	10,000–50,000	132	31.8	97	30.1	35	37.6
	50,000–10,0000	84	20.2	73	22.7	11	11.8
	100,000–200,000	44	10.6	38	11.8	6	6.5
	>200,000	36	8.7	35	10.9	1	1.1
Work status: (*n* = 412)	(1) Unemployment	210	50.6	158	49.1	52	55.9
	(2) Famer	21	5.1	17	5.3	4	4.3
	(3) Worker	15	3.6	13	4.0	2	2.2
	(4) Individual business	97	23.4	86	26.7	11	11.8
	(5) Servicer	19	4.6	11	3.4	8	8.6
	(6) Company stuff	22	5.3	17	5.3	5	5.4
	(7) Government stuff	1	0.2	1	0.3	0	0
	(8) Others	27	6.6	17	5.3	10	10.8
Months of detoxification: (*n* = 404)	(1) <1 month	50	12.0	33	10.2	17	18.3
	(2) 1–3 month	89	21.4	77	23.9	12	12.8
	(3) 3–6 month	71	17.1	48	14.9	23	24.7
	(4) 6–12 month	66	15.9	46	14.3	20	21.5
	(5) >12 month	128	30.8	109	33.9	19	20.4
Drug types	(1) heroin	108	26.0	82	25.4	26	27.6
	(2) methamphetamine	282	68.0	216	67.1	59	63.4
	(3) marihuana	9	2.2	6	1.9	3	3.2
	(4) ketamine	5	1.2	5	1.6	0	0
	(5) Morphine	3	0.7	3	0.9	0	0
	(6) MDMA (ecstasy)	3	0.7	1	0.3	2	2.2
	(7) Others	5	1.2	3	0.9	2	2.2

### Procedure

The study was approved by the Ethics Committee of Nanjing Medical University, which thoroughly considered the interests of human rights, ethics, and procedure safeties. All participants showed informed consent before involving in the study. Participants were sequentially allocated into separate meeting rooms where self-report scales took 30 min on average to ensure confidentiality. At least a research assistant was available for assisting when the participants were filing the scales.

### Measures

The present study applied the Connor–Davidson Resilience Scale [CD-RISC; (84)] for assessing the patients' resilience. The CD-RISC is a five-point Likert scale (from 0 = *not true at all* to 4 = *true nearly all the time*), which is designed to measure an individual's resilience level. The CD-RISC has 25 items with a total score ranging from 0 to 100. The scale assesses participants' optimism, strength, and toughness. The score reflects the level of resilience the individual experienced. Many studies have shown the satisfactory reliability and validity of the Chinese version of CD-RISC (85, 86). The Cronbach's α of CD-RISC was 0.906 in this study.

Positive and Negative Affect Scale (PANAS) was developed by Watson et al. (87) to evaluate individuals' positive and negative affect. The PANAS is a five-point Likert scale (from 1 = *very slight or not at all* to 5 = *very strong*) consisting of 20 items. Half of the items are subjected to the positive affect subscale (items 1, 3, 5, 9, 10, 12, 14, 16, 17, and 19). In this study, only the positive affect subscale was applied. The Chinese version of PANAS has been reported good reliability and validity (88). Cronbach's α of PANAS in the present study was 0.846.

Multidimensional Scale of Perceived Social Support [MSPSS; (89)] was used to measure participants' perceived social supports. The MSPSS incorporates three subscales, perceived family support subscale (items 3, 4, 8, and 11), perceived friend support subscale (items 6, 7, 9, and 12), and perceived specialist support subscale (items 1, 2, 5, and 10). The MSPSS is a seven-point Likert scale (from 1 = *very strongly disagree* to 7 = *very strongly agree*). The sum of items reflects the degree of an individual's overall perceived social support. The Chinese version of the MSPSS has been widely applied and showed satisfactory reliability and validity (90, 91). The Cronbach's α of the MSPSS in this study was 0.910.

Rosenberg Self-Esteem Scale [RSES; (92)] was used to assess the participants' self-esteem. The RSES is a four-point Likert scale (from 1 = *strongly disagree* to 4 = *strongly agree*), containing 10 items. The RSES was scored by summing total items after reverse-scoring negatively stated things (items 3, 6, 8, 9, and 10). Studies showed that the Chinese version of RSES has good reliability and validity (85, 93). Cronbach's α of RSES in this study was 0.656. According to (94), it is reasonable when Cronbach's coefficient is above 0.6. Although Cronbach's α of RSES is not as high as other variables, it is acceptable for the following research.

Perceived Stress Scale [PSS; (3)] was applied to measure participants' perceived stress. The PSS is a five-point Likert scale (from 0 = *Never* to 4 = *Very Often*), containing 14 items in which half of the items are positively stated (items 4, 5, 6, 7, 9, 10, and 13). The score of PPS is calculated by totaling all items after reverse-scoring the positive statement. The score reflects the level of individuals' perceived stress. The Chinese version of PSS's reliability and validity has demonstrated satisfactory consistency (95). Cronbach's α of PSS in this study was 0.729.

Satisfaction with Life Scale (SWLS) was used to evaluate an individual's life satisfaction. The SWLS includes five brief statements that can be rated by seven choices (from 1 = *strongly disagree* to 7 = *strongly agree*). The total score is measured by summing up each item. Satisfactory reliability and validity of SWLS in the Chinese population have been reported by many studies (96, 97). Cronbach's α of SWLS in this study was 0.838.

Diagnostic and Statistical Manual of Mental Disorders—fifth edition-based diagnostic questionnaires were administrated to assess participants' addiction severity. Eleven diagnostic criteria were embedded in 11 items in four categories in the questionnaire: impaired control over substance use (items 1 to 4), social consequences (items 5 to 7), risky use of the substance (items 8 to 9), and pharmacological indicators (items 10, 11). The 11 criteria include: symptoms of withdrawal, craving, tolerance, hazardous use, chronically use substantial amounts, substantial time on use, repeated attempt to abstinence, interpersonal issues related to substance use, social network collapses, absence from social and occupational events, and substance-related social and psychological issues. The addiction severity was calculated by counting the number of matched criteria. The Cronbach's α coefficient of the diagnostic question was 0.731.

### Data Analysis

In this study, sample characteristics, the descriptive statistics, and the intercorrelation analysis were measured via IBM SPSS Statistics version 22. Following Anderson and Gerbing (98), a two-step approach was used to analyze the three mediators' mediating effects. Firstly, the measurement models that contain all variables were examined by whether the indicators could well-represent each latent variable. Secondly, we use the maximum likelihood estimation to test the structural model in the AMOS 24.0 program. Furthermore, we created several parcels using the random assignment method to control the inflated measurement errors generated by multiple items of latent variables (99).

Moreover, we use AMOS 24.0 with maximum likelihood estimation to do the path analyses. According to Hu and Bentler (100) and Siedlecki et al. (101), eight indices were used to assess the goodness-of-fit of the path models: chi-square (χ2) statistics, a root-mean-square error of approximation, standardized root mean square residual, goodness-of-fit index, Tucker–Lewis index, comparative fit index, Akaike information criterion (AIC), and expected cross-validation index (ECVI). Specifically, if chi-square (χ2) statistics <3, root-mean-square error of ~ <0.08, and the upper bound of its 90% confidence interval <0.1, standardized root mean square residual <0.08, goodness-of-fit index >0.90, Tucker–Lewis index > 0.90, and comparative fit index >0.90, the model will be considered as an acceptable fit model. Furthermore, the goodness-of-fit indices of AIC and ECVI were used to compare two or more models. A smaller value of AIC and ECVI indicated a better fit of the hypothesized model (102) and a higher potential replication (103).

## Results

### Preliminary Analyses

The results of descriptive statistics (including mean, SD, Cronbach's α coefficients) and the intercorrelation analysis for all variables after considering sex and age as covariates are presented in Table 2. The results indicated that income was significantly and positively correlated with resilience, positive affect, social support, self-esteem, and life satisfaction, whereas income was negatively correlated with addiction severity. Additionally, the results suggested that addiction severity was significantly associated with resilience and positive affect. Further, all intercorrelations between resilience, positive affect, perceived social support, self-esteem, life satisfaction, and perceived stress were statistically significant.

**Table 2:** Means, standard deviations (SD), Alpha, reliabilities, and intercorrelations among study variables after controlling gender and age.

**Measure**	**Mean**	**SD**	**Alpha**	**1**	**2**	**3**	**4**	**5**	**6**	**7**	**8**
(1) Income	–	–	–	1							
(2) Addiction severity	7.30	1.94	0.731	−0.186^**^	1						
(3) Resilience	77.60	16.36	0.906	0.227^**^	−0.112^*^	1					
(4) Positive affect	25.42	7.19	0.846	0.171^**^	−0.152^**^	0.360^**^	1				
(5) Social support	52.62	13.53	0.910	0.118^*^	−0.080	0.486^**^	0.151^**^	1			
(6) Self-esteem	26.44	3.71	0.656	0.158^**^	−0.098	0.403^**^	0.226^**^	0.312^**^	1		
(7) Life satisfaction	16.45	6.55	0.838	0.160^**^	−0.081	0.278^**^	0.218^**^	0.281^**^	0.143^*^	1	
(8) Perceived stress	40.94	6.09	0.729	−0.008	0.107	−0.400^**^	−0.204^*^	−0.223^**^	−0.342^**^	−0.106^*^	1

α, Cronbach's alpha.

* Correlation is significant at the 0.05level (2-tailed).

** *Correlation is significant at the 0.01level (2-tailed)*.

According to Podsakoff et al. (104), we need to examine whether there was contamination using common method variance because self-report questionnaires measured all variables. We used the principle components factor analysis to examine a total of 76 items. The results showed 17 factors that revealed neither a single nor a general factor in this study, and the first factor would explain 19.69% of the variance. Therefore, the common method variance in this study was not a problem. Moreover, the factor analysis showed that 19 items in SWLS and PSS scales produced four factors, which indicated that the significant correlation between life satisfaction and perceived stress was not driven by method bias. The first factor explained 21.59% of the variance.

### Mediation Analyses

Without the mediator variables, the direct paths from resilience to life satisfaction (*r* = 0.278, *p* < 0.01) and to perceived stress (*r* = −0.400, *p* < 0.01) were significant. Firstly, based on the hypothesized model (Figure 1), we built Model 1 with three mediator variables (positive affect, social support, and self-esteem) with two direct paths from resilience to life satisfaction and perceived stress. The revised model suggested a satisfactory fit to the data, and all standardized path coefficients were significant, except for the three paths: positive affect to self-esteem (β = 0.005, *p* = 0.868), perceived social support to perceived stress (β = −0.013, *p* = 0.307), and self-esteem to life satisfaction (β = 0.027, *p* = 0.846; Table 3). Then, we built Model 2 by eliminating the three insignificant paths of Model 1. The test results of Model 2 were satisfactory, and all the paths were significant.

**Table 3:** Fit indices among competing models after controlling gender and age.

**Regression weights**	**Model 1**	**Model 2**	**Model 3**	**Model 4**	**Model 5**	**Target value**
χ^2^	404.810	405.877	414.259	416.683	424.961	
df	164	167	168	168	169	
χ^2^/df	2.468	2.430	2.466	2.480	2.515	<3
RMSEA	0.060 [0.052,0.067]	0.059 [0.052,0.066]	0.060 [0.052,0.067]	0.060 [0.053,0.067]	0.060 [0.053,0.068]	<0.08
SRMR	0.0619	0.0620	0.0638	0.0600	0.0619	<0.08
GFI	0.914	0.914	0.913	0.913	0.912	>0.90
TLI	0.900	0.902	0.900	0.900	0.900	>0.90
CFI	0.922	0.922	0.920	0.919	0.917	>0.90
AIC	538.810	533.877	540.259	542.683	548.961	
ECVI	1.301	1.290	1.305	1.311	1.326	

*N = 415; RMSEA, Root Mean Square Error of Approximation; SRMR, Standardized Root Mean Square Residual; []:left number, the lower bound of its 90% confidence interval <0.1; right number: the upper bound of its 90% confidence interval <0.1; GFI, Goodness-of-Fit Index; TLI, Tucker-Lewis Index; CFI, Comparative Fit Index; AIC, Akaike Information Criterion; and ECVI, Expected Cross-Validation Index*.

Then, the study tested whether mediators (positive affect, social support, and self-esteem) mediate the relationship between resilience and life satisfaction. Based on Model 2, Model 3 was built by eliminating the direct path from resilience to life satisfaction in Model 2. In Model 3, the revised model test results were satisfactory, and all the paths were significant. To compare Model 2 and Model 3, we used a chi-square difference test, which showed the model's fit decreased [Δχ2 (1, *N* = 415) = 8.382, *P* < 0.001]. Model 2, as yet, reported the best results regarding the goodness of fit.

**Figure 2:**
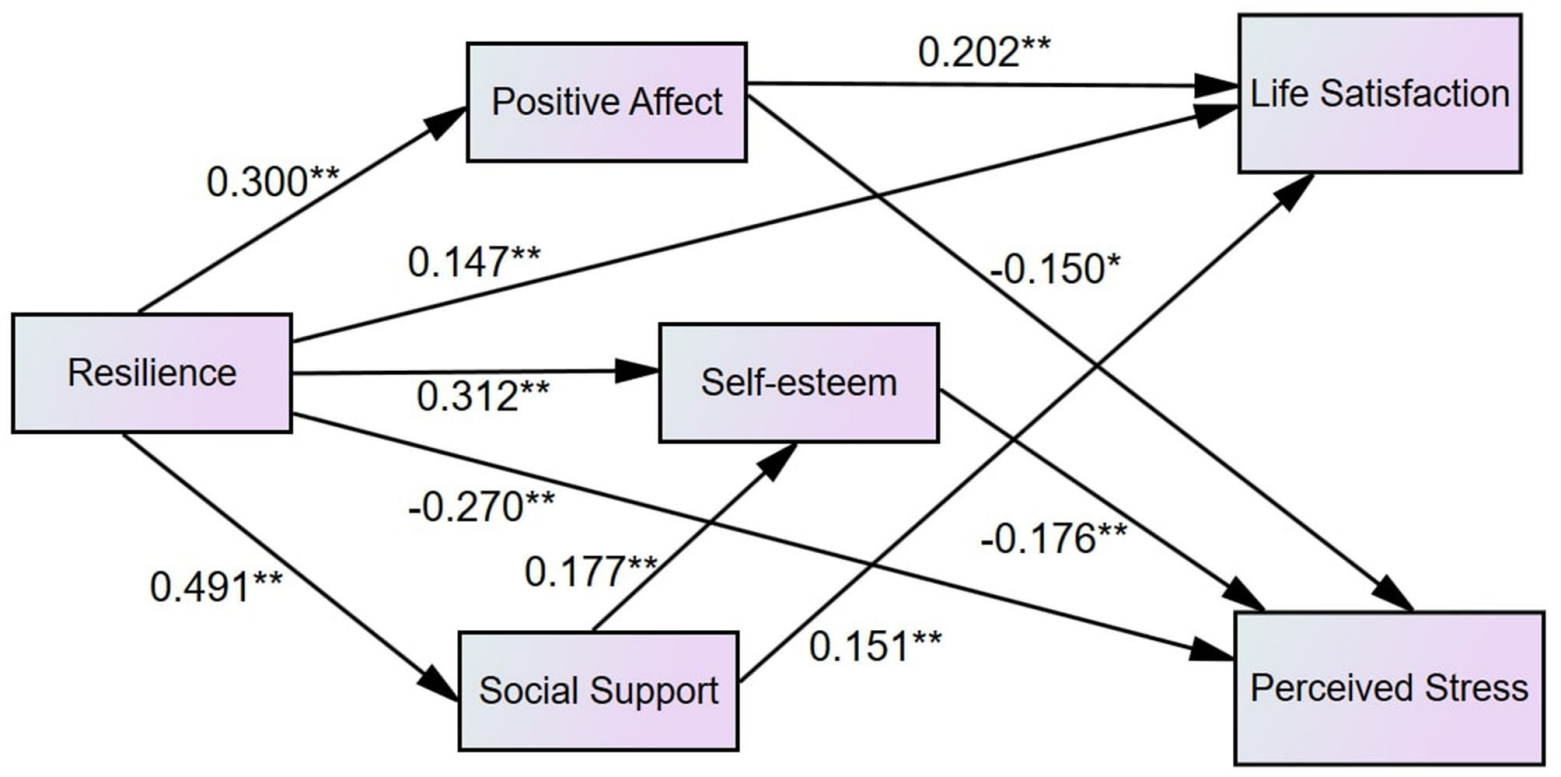
The finalized model after controlling gender and age (*N* = 415). The path coefficients are standardized. For the pictorial purpose, covariates are omitted from the figure.

Also, to test whether the mediators (positive affect, social support, and self-esteem) would mediate the relationship between resilience and perceived stress, we built Model 4 by eliminating Model 2's direct path from resilience to perceived stress. In Model 4, the revised model test results were satisfactory, and all the paths were significant. Model 5 was built by eliminating direct paths from resilience to life satisfaction and perceived stress in Model 2. The test results were also satisfactory with all the significant paths. We used a chi-square difference test to compare Model 4 with Model 5, and the results showed that the fit of the model decreased [Δχ2 (1, *N* = 415) = 8.278, *P* < 0.001]. Then, we compared the other goodness indices among five competing models, which are shown in Table 3. As a result, Model 2 was chosen as the most suitable model, and the final mediation model is shown in Figure 2.

### Indirect Effects

The indirect effects of the model were assessed by the bootstrapping procedure method in AMOS 24.0. Referring to the recommendations of (105), due to the original data set (*N* = 415), 10,000 random samples were generated after controlling the effects of sex and age. Table 4 shows the indirect effects and their corresponding 95% confidence intervals, which indicated that all the indirect effects were significant. The results supported the fact that the association between resilience and life satisfaction is partially mediated by positive affect and perceived social support through two two-path mechanisms (resilience → positive affect → life satisfaction, resilience → perceived social support → life satisfaction), and the relationship between resilience and perceived stress is partially mediated by positive affect and self-esteem through two two-path mechanisms (resilience → positive affect → perceived stress, resilience → self-esteem → perceived stress) and one three-path mechanism (resilience → perceived social support → self-esteem → perceived stress).

**Table 4:** The indirect effects of the final mediational model after controlling gender and age.

**Number**	**Model pathways**	**Point estimates**	**95%CI**	
		**β**	**Lower**	**Upper**
1	Resilience → Positive affect → Life satisfaction	0.060	0.028	0.110
2	Resilience → Social support → Life satisfaction	0.074	0.017	0.134
3	Resilience → Positive affect → Perceived stress	−0.045	−0.048	−0.027
4	Resilience → Self-esteem → Perceived stress	−0.055	−0.101	−0.021
5	Resilience → Social support → Self-esteem → Perceived stress	−0.015	−0.033	−0.005
6	Social support → Self-esteem → Perceived stress	−0.031	−0.065	−0.009

## Discussion

Numerous attempts have been made to explore how to reduce perceived stress and improve life satisfaction in non-user groups (20, 26, 28), but few focus on the individuals with SUD. To our knowledge, this is the first study designed to reveal the underlying mechanisms among resilience, perceived stress, and life satisfaction in people with SUD. We designed the study on SUD people examining whether and how resilience is associated with life satisfaction and perceived stress. The findings revealed that resilience reduces perceived stress via positive affect and self-esteem and enhances life satisfaction via positive affect and perceived stress among SUD patients.

The findings of the direct effects from resilience to perceived stress and life satisfaction demonstrated that most of the non-user groups' findings regarding the relationships among resilience, perceived stress, and life satisfaction could be replicated on people with SUD. In particular, the findings suggested that the participants who scored higher in resilience were reported to have lower perceived stress and higher life satisfaction, which are in line with the corresponding studies conducted among non-user groups (26, 28, 42). These observations may provide robust evidence for specialists and policymakers of substance abuse treatment and rehabilitation that resilience plays an effective role in mitigating perceived stress and promoting life satisfaction in substance users.

Findings also suggested that positive affect and self-esteem are two mediators of the relationship between resilience and perceived stress, supporting the study's hypothesis. These findings align with previous empirical research that resilience is negatively correlated with perceived stress (20). The theoretical underpinnings for the findings are that coping strategy promoted by resilience facilitates mental flourishing (29), the core component of positive affect, and positive self-evaluation (45), the prominent factor of self-esteem. Then, positive affect and high self-esteem promote enduring psychological resources, which may effectively buffer against the perceived stress (31). Although perceived social support's mediation effect on the relationship between resilience and perceived stress was not significant, the findings showed that perceived social support was involved in a three-path mediation (resilience → perceived social support → self-esteem → perceived stress). These findings may provide a valuable perspective on substance abuse treatment and rehabilitation. The involvement of promoting positive affect and self-esteem in rehabilitation programs among SUD patients can conditional the effects of resilience on decreasing patients' perceived stress.

The findings also indicated that the relationship between resilience and life satisfaction is mediated by positive affect and perceived social support in people with SUD, providing evidence to our hypothesis. Those findings are consistent with previous empirical research that focused on the correlations between resilience and life satisfaction (21). The theoretical interpretation of the findings is that coping strategies facilitated by resilience can stimulate the processes of psychological resource integration (106), the salient component of positive affect, and shape healthier social connections, the outstanding predictor of perceived social support (107), which further raise cognitive self-appraisals over life qualities. The findings correspond with the fact that individuals with positive affect and strong social boundaries are more easily satisfied through life events (43, 108). However, the mediation effect of self-esteem was not significant in the relationship between resilience and life satisfaction, which is opposite to the studies conducted in non-user groups (28, 109). Overall, the findings may offer an implication for SUD treatment and rehabilitation that projects focus on boosting SUD patient's life satisfaction is recommended to involve the practice of building patient's resilience, positive affect, and perceived social support.

## Conclusions

In conclusion, the present study has filled the gap in how resilience reduces perceived stress and promotes life satisfaction in SUD individuals. The study identifies that positive affect and self-esteem partially mediate the relationship between resilience and perceived stress. In contrast, the perceived social support and positive affect partially mediate the relationship between SUD patients' resilience and life satisfaction. The study may offer empirical perspectives on projecting and advancing substance abuse treatment and rehabilitation programs to reduce perceived stress and enhance life satisfaction.

## Limitations

The present study has several limitations. First, the study lacked a control group (e.g., people without SUD). Second, the present study was cross-sectional research, which is disadvantageous in drawing a causal conclusion. Therefore, involving experimental and longitudinal research methods are highly recommended in future studies. Third, given that self-report questionnaires collected all data, although measurements had shown reliability and validity, contamination cannot be entirely ignored due to social desirability (e.g., desirability for decent scores). The semi-structured interviews are recommended to be introduced in the future to reduce respondents' subjectivity. Finally, the participants' age ranged from 20 to 61 years, so it is uncertain whether the findings can be replicated in the younger and older groups. Future studies that consider these factors may generate more accurate outcomes.

## Data Availability Statement

The raw data supporting the conclusions of this article will be made available by the authors, without undue reservation.

## Ethics Statement

The studies involving human participants were reviewed and approved by the Ethics Committee of Nanjing Medical University. The patients/participants provided their written informed consent to participate in this study.

## Author Contributions

CY, YZ, and MX jointly drafted and conducted the manuscript. CY and MX contributed equally to this work. CY contributed to the processes of modeling and data analysis. YZ contributed to literature review, discussion, revision, and proofreading. MX contributed to data collection, participated in the writing, and finalized the manuscript. All authors read and approved the final manuscript.

## Conflict of Interest

The authors declare that the research was conducted in the absence of any commercial or financial relationships that could be construed as a potential conflict of interest.
